# Supplementary material for: Sertoli, Leydig, and Spermatogonial Cells’ Specific Gene and Protein Expressions as Dog Testes Evolve from Immature into Mature States
Source: Animals (Basel). 2022 Jan 22;12(3):271. doi: 10.3390/ani12030271 (PMC8833615; doi:10.3390/ani12030271)
Supplement: Supplementary file 1 [file animals-12-00271-s001.zip › animals-1535951-supplementary.pdf]

animals

Supplementary Materials

Sertoli, Leydig, and Spermatogonial Cells' Specific Gene and Protein Expressions as Dog Testes Evolve from Immature into Mature States

Vanmathy R. Kasimanickam and Ramanathan K. Kasimanickam

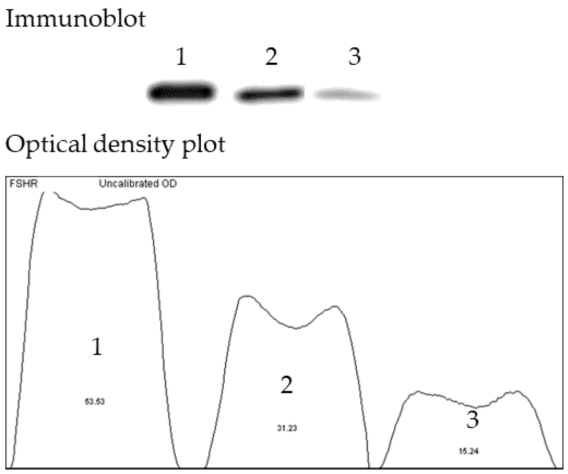

Figure S1: Representative immunoblot and optical density plot for FSHR. 1, FSHR immature testis (53.53); 2, FSHR mature testis (31.23), 3, β actin (15.24). Refer to Table 3 for mean protein expression between immature and mature testis.

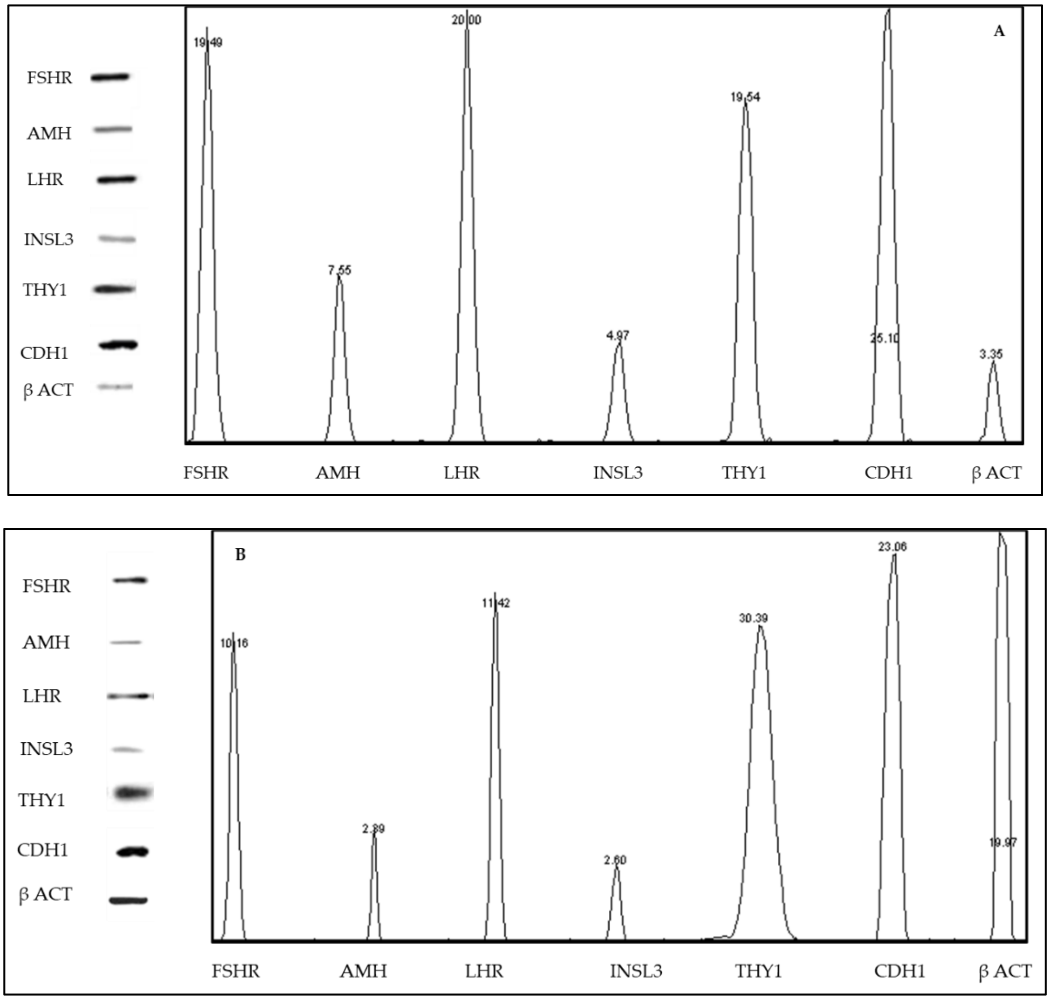

Figure S2. Representative optical density plot for proteins expression * (normalized). Quantitative analyses of relative proteins expressions in immature (**A**) and mature (**B**) canine testis Protein expressions were normalized considering all proteins.

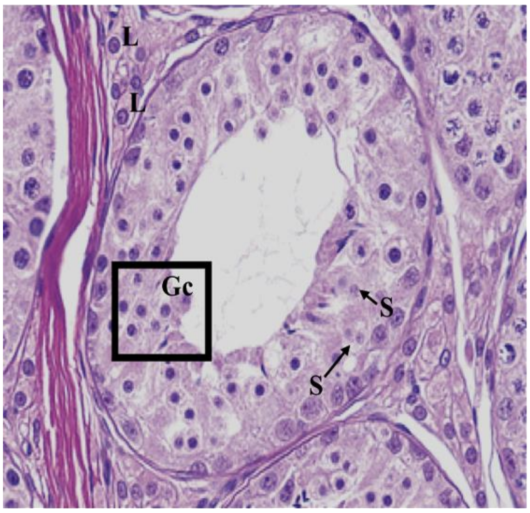

Figure S3. Sertoli cells (S) were identified in the seminiferous tubules by their pale, invaginated, irregular nuclei with a prominent nucleolus. Germ cells (Gc) are in marked in the box. Leydig cells (L) were identified in the interstitium as relatively large, ovoid-shaped cells with an eccentric nucleus containing a prominent nucleolus and peripherally localized chromatin.

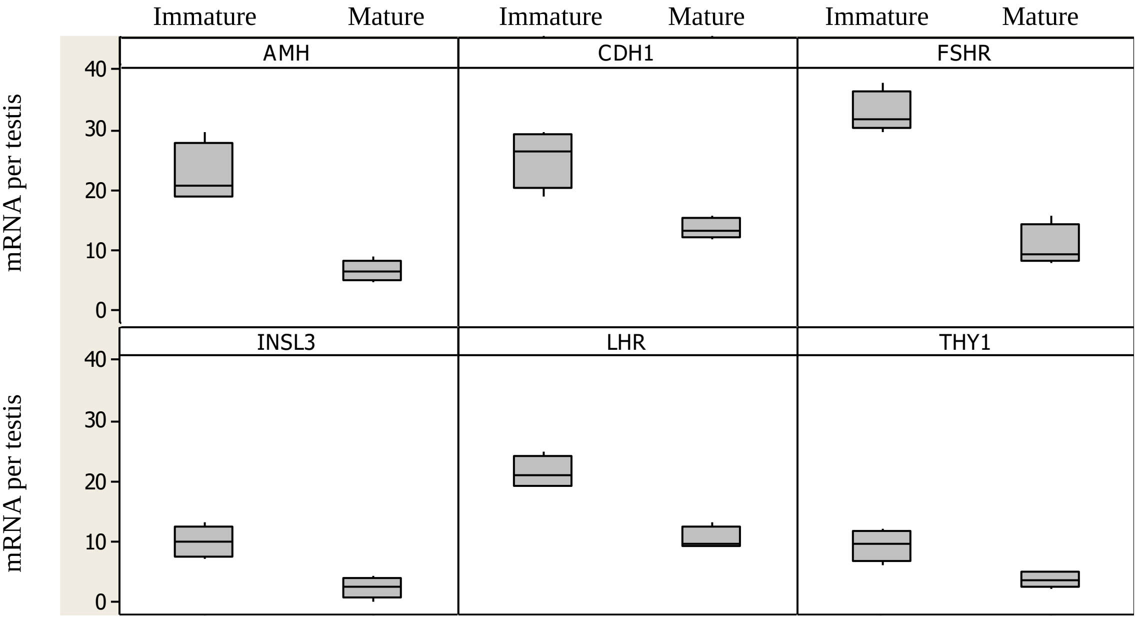

Figure S4. mRNA expression of FSHR, AMH, LHR, INSL3, THY1, and CDH1 in mature and immature testis. All genes mRNA expression differed between immature and mature dog testis ($p < 0.05$).

Table S1. Mean (± SEM) protein expressions (optical density, arbitrary units) * in immature and mature canine testis.

Protein	Immature	Mature
FSHR	19.01 ± 1.43[a]	9.16 ± 1.73[b]
AMH	8.14 ± 1.27[a]	3.45 ± 1.32[b]
LHR	21.39 ± 1.91[a]	11.42 ± 1.18[b]
INSL3	6.68 ± 1.15[a]	2.22 ± 1.21[b]
THY1	24.22 ± 1.54[a]	19.83 ± 1.33[b]
CDH1	26.67 ± 1.93[a]	19.11 ± 1.24[b]

a,b, Values with different superscripts between mature and immature testis are different ($p < 0.05$). * Normalized considering all protein in immature and in mature testis (endogenous control, β actin).
